# Supplementary material for: Cathelicidin Antimicrobial Peptide Levels in Atherosclerosis and Myocardial Infarction in Mice and Human
Source: Int J Mol Sci. 2024 Mar 2;25(5):2909. doi: 10.3390/ijms25052909 (PMC10931542; doi:10.3390/ijms25052909)
Supplement: Supplementary file 1 [file ijms-25-02909-s001.zip › ijms-2871545-supplementary.pdf]

Supplementary data

CAMP [ng/mL]	Total cohort (n=234)	Ctrl. (n=31)	CAD (n=203)	CCS (n=64)	ACS-1 (n=73)	ACS-2 (n=66)
age [y]	n.s.	n.s.	n.s.	n.s.	n.s.	n.s.
height [cm]	n.s.	n.s.	n.s.	n.s.	n.s.	n.s.
weight [kg]	n.s.	n.s.	n.s.	n.s.	n.s.	n.s.
BMI [kg/m²]	n.s.	n.s.	n.s.	n.s.	n.s.	rho=0.266 p=0.031* n=66
BP systolic [mmHg]	n.s.	n.s.	rho=0.155 p=0.037* n=180	n.s.	n.s.	n.s.
BP diastolic [mmHg]	rho=0.177 p=0.010* n=210	n.s.	rho=0.182 p=0.014* n=180	n.s.	n.s.	n.s.
Total cholesterol [mg/dL]	n.s.	n.a.	n.s.	n.s.	n.s.	n.s.
LDL cholesterol [mg/dL]	n.s.	n.a.	n.s.	n.s.	n.s.	n.s.
HDL cholesterol [mg/dL]	n.s.	n.a.	n.s.	n.s.	n.s.	n.s.
Triglycerides [mg/dL]	rho=0.181 p=0.023* n=158	n.a.	rho=0.176 p=0.028* n=156	rho=0.514 p<0.001* n=41	n.s.	n.s.
Leukocytes [giga/L]	n.s.	n.a.	n.s.	rho=0.323 p=0.013* n=59	n.s.	n.s.
Monocytes [% total monocytes]	n.s.	n.s.	n.s.	n.s.	n.s.	n.s.
$CD14^{++}/CD16^{-}$ [% total monocytes]	n.s.	n.s.	n.s.	n.s.	n.s.	n.s.
$CD14^{++}/CD16^{+}$ [% total monocytes]	n.s.	n.s.	n.s.	n.s.	n.s.	n.s.
$CD14^{+}/CD16^{++}$ [% total monocytes]	n.s.	n.s.	n.s.	n.s.	n.s.	n.s.
CRP [mg/L]	n.s.	n.a.	n.s.	n.s.	n.s.	n.s.
CTRP-3 [ng/mL]	n.s.	n.s.	n.s.	n.s.	n.s.	n.s.
IL-1β [pg/mL]	n.s.	rho=0.461 p=0.041* n=20	n.s.	n.s.	n.s.	rho=0.446 p=0.033* n=23
IL-6 [pg/mL]	n.s.	n.s.	n.s.	n.s.	n.s.	n.s.
TNF-a [pg/mL]	n.s.	n.s.	n.s.	n.s.	n.s.	n.s.
MCP-1 [pg/mL]	rho=0.239 p=0.026* n=86	n.s.	n.s.	n.s.	rho=-0.512 p=0.030* n=18	n.s.
BNP [pg/mL]	n.s.	n.a.	n.s.	n.s.	n.s.	n.s.
Tni [µg/L]	n.s.	n.a.	n.s.	n.s.	n.s.	n.s.
LV-EF [%]	n.s.	n.a.	n.s.	n.s.	n.s.	rho=-0.376 p=0.026* n=35

Supplementary Table S1: Correlation analyses in the subgroups with regards to CAD.
Correlation analyses were performed with CAMP and with anthropometric, inflammatory, metabolic, and myocardial function parameters for the entire study cohort and as well as in subgroups defined by coronary artery disease. CAMP serum levels were measured by ELISA. Spearman-rho test was applied for calculation of correlation coefficient and statistical significance. n.a.: not available; n.s.: not significant; CAMP: Cathelicidin antimicrobial peptide; Ctrl.: Control; CAD: coronary artery disease; CCS: chronic coronary syndrome; ACS: acute coronary syndrome; BMI: body mass index; BP: blood pressure; LDL: low-density lipoprotein; HDL: high-density lipoprotein; CRP: C-reactive peptide; CTRP-3: C1q/TNF-Related Protein 3; IL: Interleukin; TNF-a: tumor necrosis factor a; MCP-1: monocyte chemoattractant protein-1; BNP: brain natriuretic peptide; LV-EF: left vetricular ejection fraction. * $p < 0.05$

CAMP [ng/mL]	men<br><br>(n=177)	men<br>Ctrl.<br>(n=17)	men<br>CAD<br>(n=160)	women<br><br>(n=57)	women<br>Ctrl.<br>(n=14)	women<br>CAD<br>(n=43)
age [y]	n.s.	n.s.	n.s	rho=0.261<br>p=0.050*<br>n=57	n.s.	n.s.
height [cm]	n.s.	n.s.	n.s.	n.s.	n.s.	n.s.
weight [kg]	n.s.	n.s.	n.s.	rho=0.315<br>p=0.017*<br>n=57	n.s.	rho=0.402<br>p=0.007*<br>n=43
BMI [kg/m²]	n.s.	n.s.	n.s.	n.s.	n.s.	n.s.
BP systolic [mmHg]	n.s.	n.s.	n.s.	rho=0.324<br>p=0.019*<br>n=52	n.s.	rho=0.325<br>p=0.047*<br>n=38
BP diastolic [mmHg]	n.s.	n.s.	n.s.	n.s.	n.s.	n.s.
Total cholesterol [mg/dL]	n.s.	n.a.	n.s.	n.s.	n.a.	n.s.
LDL cholesterol [mg/dL]	n.s.	n.a.	n.s.	n.s.	n.a.	n.s.
HDL cholesterol [mg/dL]	n.s.	n.a.	n.s.	n.s.	n.a.	n.s.
Triglycerides [mg/dL]	rho=0.200<br>p=0.026*<br>n=125	n.a.	rho=0.200<br>p=0.026*<br>n=125	n.s.	n.a.	n.s.
Leukocytes [giga/L]	rho=0.163<br>p=0.047*<br>n=149	n.a.	rho=0.163<br>P=0.047*<br>n=149	n.s.	n.a.	n.s.
Monocytes [% total monocytes]	n.s.	n.s.	n.s.	n.s.	n.s.	n.s.
$CD14^{++}/CD16^{-}$ [% total monocytes]	n.s.	n.s.	n.s.	rho=-0.292<br>p=0.038*<br>n=51	n.s.	n.s.
$CD14^{++}/CD16^{+}$ [% total monocytes]	n.s.	n.s.	n.s.	n.s.	n.s.	n.s.
$CD14^{+}/CD16^{++}$ [% total monocyte]	n.s.	n.a.	n.s.	n.s.	n.s.	n.s.
CRP [mg/L]	n.s.	n.a.	n.s.	n.s.	n.a.	n.s.
CTRP-3 [ng/mL]	n.s.	n.s.	n.s.	n.s.	n.s.	n.s.
IL-1β [pg/mL]	rho=0.297<br>p=0.013*<br>n=69	n.s.	n.s.	n.s.	n.s.	n.s.
IL-6 [pg/mL]	n.s.	n.s.	n.s.	n.s.	n.s.	n.s.
TNF-a [pg/mL]	n.s.	n.s.	n.s.	n.s.	n.s.	n.s.
MCP-1 [pg/mL]	rho=0.249<br>p=0.039*<br>n=69	n.s.	n.s.	n.s.	n.s.	n.s.
BNP [pg/mL]	n.s.	n.a.	n.s.	n.s.	n.a.	n.s.
Tni [µg/l]	n.s	n.a.	n.s.	n.s.	n.a.	n.s.
LV-EF [%]	n.s.	n.a.	n.s.	n.s.	n.a.	n.s.

Supplementary Table S2: Correlation analyses in subgroups with regards to gender

Correlation analyses were performed with CAMP and with anthropometric, inflammatory, metabolic, and cardiac function parameters for the entire study cohort as well as in men and women separately. CAMP serum concentrations were measured by ELISA. Spearman-rho test was applied for calculation of correlation coefficient and statistical significance. n.a.: not available; n.s.: not significant; CAMP: Cathelicidin antimicrobial peptide; Ctrl.: Control; CAD: coronary artery disease; BMI: body mass index; BP: blood pressure; LDL: low-density lipoprotein; HDL: high-density lipoprotein; CRP: C-reactive peptide; CTRP-3: C1q/TNF-Related Protein 3; IL: Interleukin; TNF-a: tumor necrosis factor a; MCP-1: monocyte chemoattractant protein-1; BNP: brain natriuretic peptide; LV-EF: left vetricular ejection fraction. * $p < 0.05$

CAMP [ng/mL]	lean (n=47)	lean Ctrl. (n=10)	lean CAD (n=37)	obese (n=185)	obese Ctrl. (n=21)	obese CAD (n=164)
age [y]	n.s.	n.s.	n.s.	n.s.	n.s.	n.s.
height [cm]	n.s.	n.s.	n.s.	n.s.	n.s.	n.s.
weight [kg]	n.s.	n.s.	n.s.	n.s.	n.s.	n.s.
BMI [kg/m²]	n.s.	n.s.	n.s.	n.s.	n.s.	n.s.
BP systolic [mmHg]	n.s.	n.s.	n.s.	n.s.	n.s.	n.s.
BP diastolic [mmHg]	n.s.	n.s.	n.s.	rho=0.202 p=0.010* n=162	n.s.	rho=0.198 p=0.018* n=142
Total cholesterol [mg/dL]	n.s.	n.a.	n.s.	n.s.	n.a.	n.s.
LDL cholesterol [mg/dL]	n.s.	n.a.	n.s.	n.s.	n.a.	n.s.
HDL cholesterol [mg/dL]	n.s.	n.a.	n.s.	n.s.	n.a.	n.s.
Triglycerides [mg/dL]	n.s.	n.a.	n.s.	n.s.	n.a.	n.s.
Leukocytes [giga/L]	n.s.	n.a.	n.s.	n.s.	n.a.	n.s.
Monocytes [% total monocytes]	n.s.	rho=-0.667 p=0.050 n=9	n.s.	n.s.	n.s.	n.s.
$CD14^{++}/CD16^{-}$ [% total monocytes]	rho=-0.380 p=0.010* n=45	n.s.	rho=-0.376 p=0.024* n=36	n.s.	n.s.	n.s.
$CD14^{++}/CD16^{+}$ [% total monocytes]	n.s.	n.s.	n.s.	n.s.	n.s.	n.s.
$CD14^{+}/CD16^{++}$ [% total monocytes]	n.s.	n.s.	n.s.	n.s.	n.s.	n.s.
CRP [mg/L]	n.s.	n.a.	n.s.	n.s.	n.a.	n.s.
CTRP-3 [ng/mL]	n.s.	n.s.	n.s.	n.s.	n.s.	n.s.
IL-1β [pg/mL]	n.s.	n.s.	n.s.	n.s.	n.s.	n.s.
IL-6 [pg/mL]	n.s.	n.s.	n.s.	n.s.	n.s.	n.s.
TNF-a [pg/mL]	n.s.	n.s.	n.s.	n.s.	n.s.	n.s.
MCP-1 [pg/mL]	n.s.	n.s.	n.s.	n.s.	n.s.	n.s.
BNP [pg/mL]	n.s.	n.a.	n.s.	n.s.	n.a.	n.s.
TNI [µg/L]	n.s.	n.a.	n.s.	n.s.	n.a.	n.s.
LV-EF [%]	n.s.	n.a.	n.s.	n.s.	n.a.	n.s.

Supplementary Table S3: Correlation analyses in subgroups with regards to BMI

Correlation analyses were performed with CAMP and with anthropometric, inflammatory, metabolic, and cardiac function parameters for patients regarding their BMI. CAMP serum concentrations were measured by ELISA. Spearman-rho test was applied for calculation of correlation coefficient and statistical significance. n.a.: not available; n.s.: not significant; lean: BMI < 25kg/m²; obese: BMI ≥ 25kg/m²; CAMP: Cathelicidin antimicrobial peptide; Ctrl.: Control; CAD: coronary artery disease; BMI: body mass index; BP: blood pressure; LDL: low-density lipoprotein; HDL: high-density lipoprotein; CRP: C-reactive peptide; CTRP-3: C1q/TNF-Related Protein 3; IL: Interleukin; TNF-a: tumor necrosis factor a; MCP-1: monocyte chemoattractant protein-1; BNP: brain natriuretic peptide; LV-EF: left vetricular ejection fraction. * $p < 0.05$

**A** CAMP [ng/mL]	Total cohort (n=234)	Ctrl. (n=31)	CAD (n=203)	CCS (n=64)	ACS-1 (n=73)	ACS-2 (n=66)
sex	n.s.	n.s.	n.s.	n.s.	n.s.	n.s.
BMI	n.s.	n.s.	n.s.	n.s.	n.s.	n.s.
NYHA	n.s.	n.s.	n.s.	n.s.	n.s.	n.s.
Diabetes mellitus	n.s.	n.s.	n.s.	0.012*	n.s.	n.s.
Hypertension	n.s.	n.s.	n.s.	n.s.	n.s.	n.s.
Dyslipidaemia	n.s.	n.s.	n.s.	n.s.	n.s.	n.s.
Family history	n.s.	n.s.	n.s.	n.s.	n.s.	n.s.
Smoker	n.s.	n.s.	n.s.	n.s.	n.s.	n.s.

**B** CAMP [ng/mL]	men (n=177)	Ctrl. (n=17)	CHD (n=160)	women (n=57)	Ctrl. (n=14)	CHD (n=43)
BMI	n.s.	n.s.	n.s.	n.s.	n.s.	n.s.
NYHA	n.s.	n.s.	n.s.	0.021*	n.s.	n.s.
Diabetes mellitus	n.s.	n.s.	n.s.	n.s.	n.s.	n.s.
Hypertension	n.s.	n.s.	n.s.	n.s.	0.038*	n.s.
Dyslipidaemia	n.s.	n.s.	n.s.	n.s.	n.s.	n.s.
Family history	n.s.	n.s.	n.s.	n.s.	n.s.	n.s.
Smoker	n.s.	n.s.	n.s.	n.s.	n.s.	n.s.

**C** CAMP [ng/mL]	lean (n=47)	Ctrl. (n=10)	CHD (n=37)	obese (n=185)	Ctrl. (n=21)	CHD (n=164)
sex	n.s.	n.s.	n.s.	n.s.	n.s.	n.s.
NYHA	n.s.	n.s.	n.s.	n.s.	n.s.	n.s.
Diabetes mellitus	n.s.	n.s.	n.s.	n.s.	n.s.	n.s.
Hypertension	n.s.	n.s.	n.s.	n.s.	n.s.	n.s.
Dyslipidaemia	n.s.	n.s.	n.s.	n.s.	n.s.	n.s.
Family history	n.s.	n.s.	n.s.	n.s.	n.s.	n.s.
Smoker	n.s.	n.s.	n.s.	n.s.	n.s.	n.s.

Supplementary Table S4: Comparison of mean CAMP concentrations

Mean systemic CAMP concentrations were investigated with respect to cardiovascular risk factors within subgroups defined by coronary artery disease (A), gender (B), and BMI (C). CAMP serum concentrations were measured by ELISA. Comparison of means were performed with Man-Whitney or Kruskal-Wallis test for calculation of statistical significance. n.s.: not significant; Ctrl: Control; CAD: coronary artery disease; CCS: chronic coronary syndrome; ACS: acute coronary syndrome; lean: BMI < 25kg/m²; obese: BMI ≥ 25kg/m²; BMI: body mass index; NYHA: Classification of the New York Heart Association.
